# Peripheral Ossifying Fibroma in the Oral Cavity: MRI Findings

**DOI:** 10.1155/2011/190592

**Published:** 2011-10-20

**Authors:** Swapnali Chaudhari, Hemant R. Umarji

**Affiliations:** Department of Oral Medicine and Radiology, Government Dental College and Hospital, Mumbai 1, India

## Abstract

The peripheral ossifying fibroma, a benign gingival overgrowth, occurs mainly in the anterior portion of the maxilla in young adults. The size of the lesion is usually less than 1.5 cm. We report a case of peripheral ossifying fibroma overlying the mandibular alveolar ridge of a 55-year-old female. MR scans showed a large exophytic soft tissue mass overlying mandibular alveolar ridge. The tumor revealed peripheral calcifications with slight erosion of adjacent cortical plate.

## 1. Introduction

The peripheral ossifying fibroma (POF), sometimes referred to as fibrous epulis, calcifying fibroblastic granuloma, or peripheral fibroma with calcification, is a localized reactive enlargement of the gingiva that typically measures less than 1.5 cm at its greatest dimensions. The POF may appear ulcerated and erythematous or exhibit a color similar to the surrounding gingiva. It may be pedunculated or sessile and does not blanch upon palpation [1]. Histologically, the POF is a noncapsulated mass of a very cellular fibroblastic connective tissue covered by stratified squamous epithelium. Randomly distributed calcifications may be dispensed throughout the cellular connective tissue [2].

To our knowledge, in the radiology literature, only one article has described CT and MRI features of peripheral ossifying fibroma [3]. We report MRI findings of unusually large POF.

## 2. Case Report

**Figure 1:**
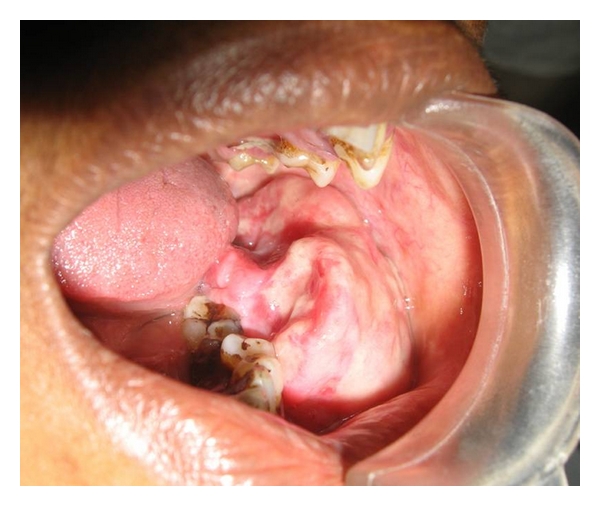
Intraoral photograph.

A 55-year-old female presented with an exophytic mass in the oral cavity that had enlarged gradually for 6 months. Extraorally, there was a facial asymmetry due to bulging of the left cheek. Intraoral examination revealed poor oral hygiene and neglected dental condition. A pedunculated, rubbery, nontender, and pinkish mass of gingival-like tissue was seen extending from lower left first premolar to lower left second molar, occupying entire lower left buccal vestibule (Figure 1). It measured 5.9 cm in diameter. Soft tissue radiograph of the same region showed calcifications within the soft tissue mass.

**Figure 2:**
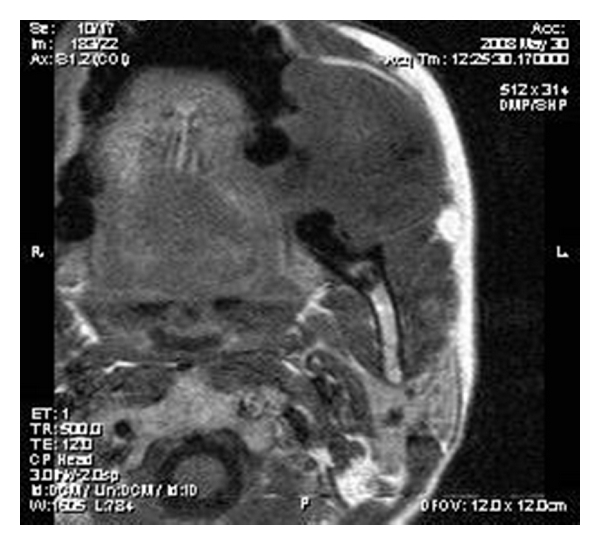
T_1_-weighted axial image showing well-marginated isointense soft tissue lesion with few hypointense areas within.

**Figure 3:**
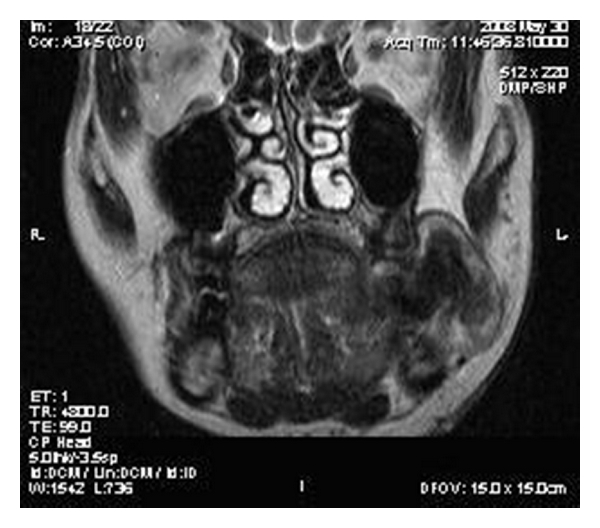
T_1_-weighted coronal image showing isointense mass occupying left vestibule.

**Figure 4:**
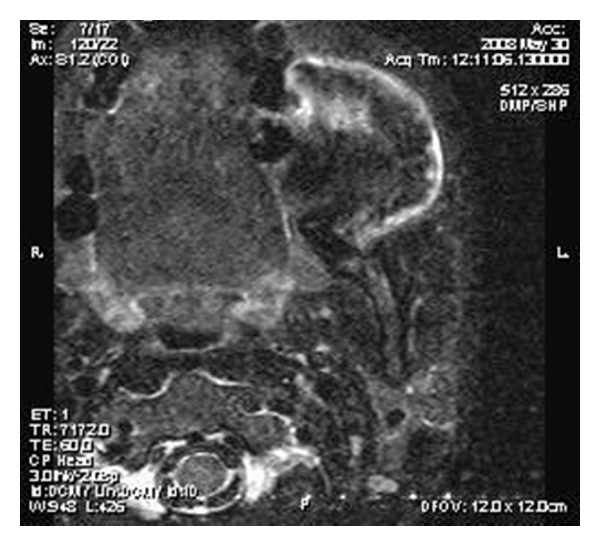
T_2_-weighted axial image showing iso- to hyperintense mass.

**Figure 5:**
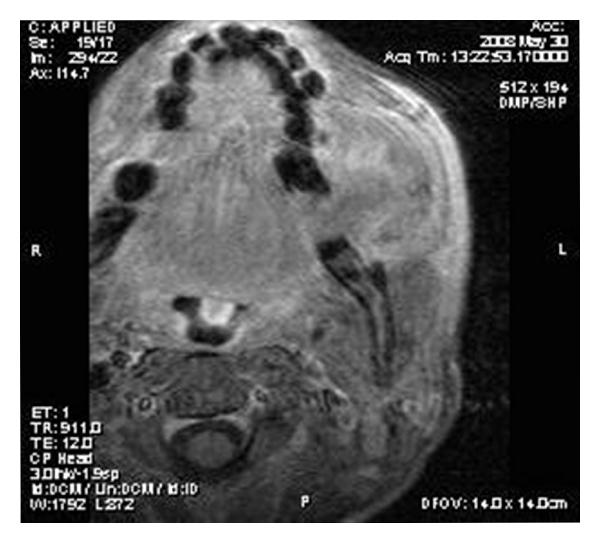
Postcontrast T_1_-weighted axial image showing heterogenous enhancement.

On MR imaging, the lesion was homogenously isointense on nonenhanced T_1_-weighted sequences (Figures 2 and 3) and iso- to hypointense on T_2_-weighted sequences (Figure 4). Multiple small hypointense areas were seen within the mass on nonenhanced T_1_-weighted sequences suggestive of calcifications. The mass had caused displacement of zygomaticus major muscle and facial artery, but the fat planes were preserved. Slight erosion of adjacent cortical plate was noted as loss of hypointensity of cortical plate adjacent to the mass on nonenhanced T_1_-weighted sequences. After administration of contrast (gadolinium diethylene triamine pentaacetic acid), the lesion showed heterogenous enhancement (Figure 5).

**Figure 6:**
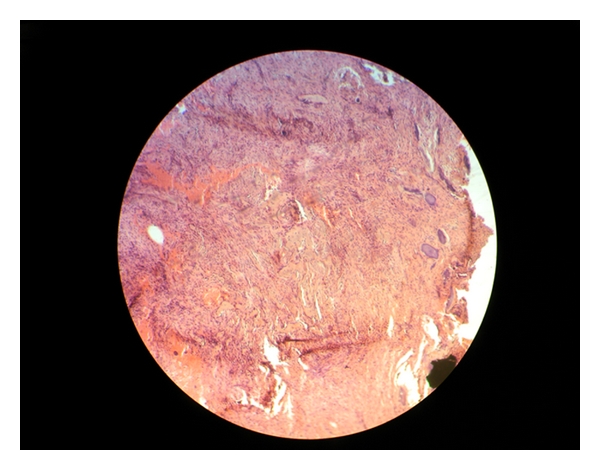
Photomicrograph confirming the diagnosis of peripheral ossifying fibroma.

Total excision of the lesion was then performed. Histopathology report showed fibrocellular connective tissue stroma with plump proliferating fibroblasts. Stroma showed areas of osteoid and immature bone tissue surrounded by the plump proliferating fibroblasts. Small round cementum-like material was also evident at few areas. Pathological examination confirmed the diagnosis of a peripheral ossifying fibroma (Figure 6). 

## 3. Discussion

The POF occurs almost exclusively on the free margin of gingiva and usually involves the interdental papilla. While its etiology is unclear, POFs are frequently associated with irritants like calculus, plaque, dental appliances, ill-fitting crowns, and rough restorations. Inflammatory hyperplasia originating in the superficial periodontal ligament is considered to be a factor in the histogenesis of the POF. POF can occur at any age but exhibits a peak incidence between second and third decades. Females are affected more than males. 60% of the lesions occur in the maxilla, with more than 50% occurring in the incisor-canine region [4]. Clinically, it presents as an exophytic, smooth-surfaced, pink or red nodular mass that may be sessile or pedunculated. Interdental gingival papilla is frequently involved. Most of the reported POFs have been 1-2 cm in size [3]. 

The clinical differential diagnosis includes peripheral giant cell granuloma (PGCG), pyogenic granuloma, fibroma, peripheral odontogenic fibroma, hemangioma, and chondrosarcoma or osteosarcoma [5]. Pyogenic granuloma is highly vascular nontumorous condition involving gingival tissues with a tendency to hemorrhage. They are usually small and only occasionally show calcifications. It is possible to histologically differentiate PGCG and peripheral odontogenic fibroma from POF as PGCG contains giant cells whereas peripheral odontogenic fibroma contains odontogenic epithelium and dysplastic dentine; all the features not seen in POF [6]. A majority of hemangiomas are congenital, but some are acquired later in life. Some of the acquired capillary hemangiomas of the oral cavity may develop from inflammatory hyperplasia lesions mostly on the gingiva. The lesion is usually nodular and bluish red, bleeds easily, and may blanch on pressure. Also radiographically phleboliths may be seen. Chondrosarcoma and osteosarcoma are less frequent gingival lesions. Although slight bony resorption may occur beneath POF, more worrisome bony changes typically are seen with malignant lesions. A band-like asymmetric widening of the PDL of involved teeth is another important finding suggestive of osteosarcoma or chondrosarcoma [5].

A peripheral ossifying fibroma is usually small and does not require any further imaging study in addition to plain radiographs. In this case, the size of the lesion reached 5.9 cm, and it was a soft tissue lesion with minimum bone involvement; hence, MRI study was considered.

On MR imaging, the lesion was isointense on nonenhanced T_1_-weighted sequences, iso- to hypointense on T_2_-weighted sequence, and showed heterogenous postcontrast enhancement. Isointense nature on T_2_-weighted sequence implies hypercellularity or lesion with high nuclear cytoplasmic ratio. All the fat planes were preserved, and there was displacement rather than destruction of adjacent anatomical structures. This finding suggests the benign nature of the lesion. Hemangiomas are hyperintense on T_2_-weighted sequences. The marked high signal intensity on T_2_ is related to the increased proportion of free water within the stagnant blood found in large vessels of the lesion [7]. Pyogenic granulomas are highly vascular, and hence are again hyperintense on T_2_-weighted sequences. Though calcifications are seen better on CT, they were well appreciated as multiple small hypointense areas on nonenhanced T_1_-weighted sequences.

This is a unique case of large peripheral ossifying fibroma overlying mandibular alveolar ridge. MRI study was helpful in the diagnosis of the present case.
